# Cohort studies of Faroese children concerning potential adverse health effects after the mothers’ exposure to marine contaminants during pregnancy

**DOI:** 10.1186/1751-0147-54-S1-S7

**Published:** 2012-02-24

**Authors:** Pál Weihe, Philippe Grandjean

**Affiliations:** 1 The Faroese Hospital System, Department of Occupational and Public Health, Sigmundargøta 5, FO-110 Tórshavn, Faroe Islands; 2 University of Southern Denmark, Institute of Public Health, Department of Environmental Medicine, Winsloewsparken 17, 5000 Odense C, Denmark

## Background

Toxicological evidence suggests that humans are much more vulnerable to adverse effects from exposures to pollutants that occur during development, i.e., prenatally or in early childhood. However, the adverse effects may not be immediately apparent and often are expressed fully only when physiological functions have matured. Accordingly, research in environmental epidemiology now emphasizes prospective research, in this case based on birth cohorts. Given the advantages of conducting such research in the Faroe Islands, we have therefore generated five birth cohorts. In addition, we have used available records on whaling during the past century to clarify prenatal methylmercury exposure of elderly people on the basis of availability.

### Cohort 1

A cohort of 1022 singleton births was assembled in the Faroe Islands during a 21-month period of 1986-1987. The range of mercury concentrations in cord blood and maternal hair was about 1000-fold. Frequent whale meat dinners during pregnancy and, to a much lesser degree, frequent consumption of fish, and increased parity or age were associated with high mercury concentrations in cord blood and maternal hair. Blood-mercury levels were slightly lower if the mother had ingested alcoholic beverages (which happened only occasionally in this group). Mercury in cord blood correlated moderately with blood-selenium. Lead in cord blood was low (median, 82 nmol/l), particularly when the mothers had frequently had fish for dinner and abstained from smoking. Because the effects of fetal childhood exposure to methylmercury are persistent, detailed examination of children with prenatal exposure to this neurotoxicant would be appropriate at school age. At this time, they have developed sufficiently to perform a wide variety of neurobehavioral tests, and they are capable of cooperating for most functional tasks. The first detailed examination took place at age 7 years, i.e., just before school entry, between early April and late June in 1993 and, for the youngest children of the cohort, at the same time in 1994. Children currently residing in Denmark were examined in 1994. A total of 917 of the surviving children (90.3%) completed the examinations. Because of a slightly lower participation rate of children from the capital of Torshavn, the prenatal mercury exposure levels of the children examined was significantly higher [geometric mean cord-blood mercury concentration, 22.8 µg/l (114 nmol/l)] than that of those who did not participate [17.9 µg/l (89 nmol/l)]. Because no other selection bias was apparent, the small attrition would be unlikely to affect a relationship between mercury exposure and neurobehavioral function. Most of the children were examined at the National Hospital in Tórshavn, the capital of the Faroe Islands. All transportation costs were refunded. Four children were examined during the morning and four during the afternoon at five examination stations, with each station taking up to 60 minutes. Past medical history, current health status and social factors were recorded on a self-administered form by the parent accompanying the child (usually the mother). The physical examination included a functional neurological examination with emphasis on motor coordination and perceptual-motor performance. Visual acuity was determined by Snellen's board and contrast sensitivity by the Functional Acuity Contrast Test. Otoscopy and tympanometry were supplemented by audiometry. Main emphasis was placed on detailed neurophysiological and neuropsychological tests that had been selected on the basis of a range of considerations. Tests were chosen to include tasks that would be affected by the neuropathological abnormalities described in congenital methylmercury poisoning and the functional deficits seen in children with early-life exposure to neurotoxicants. The tests also had to be acceptable to the children and their parents, viz. painless, not too time-consuming, and appropriate for 7-year-old Faroese children who had not yet begun school. Tests that were likely to provide a high statistical sensitivity, i.e., with a wide range of scores possible without floor or ceiling effects, and acceptable test-retest reliability, were preferred. In addition, test versions standardized in Scandinavian countries were favored. The second examinations were completed at age 14 years. Again, the participation rate was very high, almost 90%. The overall approach was very similar to the one previously applied, though the clinical tests were adjusted to be appropriate for the teenage participants. Likewise, comprehensive examinations were carried out at age 23 years. As many cohort members had moved to Denmark for education or employment, a clinic was also established in Copenhagen. Educational achievement is being evaluated using the results from the standardized tests at completion of the 9th grade and the status at age 22 years.

### Cohort 2

The findings from Cohort 1 suggested that exposure assessment should encompass several lipophilic pollutants in addition to methylmercury. As a follow-up, Cohort 2 was therefore established during a 12-month period in 1994-1995 and included 182 singleton term births from consecutive births at the National Hospital in Tórshavn, Faroe Islands. Maternal residence was required in the central and northwestern region of the primary catchment area, i.e., away from the capital area of Tórshavn. About one-third of the Faroese population resides in this area, where the mercury exposure was expected to vary the most. A total 64% of all births were included, incomplete sampling being mainly due to logistic problems in the busy ward. The overall participation rate was slightly below the one obtained in Cohort 1, but the average birth weight was almost the same in the two cohorts and similar to the Faroese average. Relevant obstetric data were obtained by standardized procedures and supplemented by a brief nutrition questionnaire. These children were first examined by the Neurological Optimality Score at age two weeks (adjusted for gestational age), and then again at 7 months of age. Subsequent examinations were at age 18 months and then at 12-month intervals up to age 66 months. At 42 months, a comprehensive medical examination with the Neurological Optimality Score was included. For comparison with Cohort 1, detailed neurobehavioral tests were carried out at age 7 years. A repeat examination was then completed at age 10 years. The complete profile of neurobehavioral development is currently being analyzed. This cohort also participated in a study of serum antibody concentrations as a measure of the effects of routine childhood immunizations.

### Cohort 3

New insight into health risks caused by environmental pollutants and changing exposure patterns in the Faroes lead to the formation of Cohort 3 from consecutive births in Tórshavn between 1 April, 1998 and 29 February, 2000. Because of dietary recommendations from the Faroese health authorities, methylmercury exposures had now decreased thus allowing better characterization of possible effects of PCBs and other lipophilic contaminants. The main part of Cohort 3 consists of 547 children. Inclusion criteria required appropriate biological specimens for exposure biomarker determination and a valid examination by the pediatrician at two weeks of age. The children included represent approximately 60% of all pregnancies. Most of the attrition was caused by work schedules in the busy ward or scheduling problems. Cohort members have a slightly greater birth weight and slightly older mothers with a somewhat greater parity as compared to non-responders. On the other hand, the child's sex and Apgar score are similar in participants and non-participants. In regard to parity, maternal age, smoking and alcohol consumption (very limited), Cohort 3 is quite similar to the two previously generated cohorts. Serum was again collected from the mother at the last antenatal examination (34th week of pregnancy). Other samples collected from the mother-child pairs include cord blood and serum, maternal hair at parturition, and milk on days 3-5 (before mother and child were released) and at two weeks. Nutritional habits were recorded by questionnaire (number of whale meat dinners per month during pregnancy and before pregnancy; number of fish dinners per week; ingestion of blubber with whale meat or fish). A subset of 150 mothers also filled in a detailed food frequency questionnaire. A subgroup of Cohort children was examined with regard to immunological parameters at ages 11 and 18 months. The first comprehensive medical examination was carried out just before the booster vaccination at age 5 years, with a follow-up blood sample one month after vaccination. The children were again examined at age 7 years, with a main focus on immunological parameters. A pre-puberty examination is planned for age 12 years to follow the long-term implications of the immunotoxicity changes already documented.

### Cohort 4

Subsequent to the decision, in August 1998, by Faroese health authorities to recommend that women should reduce their intake of pilot whale meat and blubber – in order to protect the foetus against adverse effects from food contaminants – we carried out a study to determine the effects of this recommendation. The data collection took place during 12 months from October 2000, and 148 women (49.7% of all eligible) completed the full protocol. Blood samples from the 37th week of pregnancy were analyzed for heavy metals and organochlorine compounds. In addition, we used a 24-hour recall as well as a food diary, and also a food frequency questionnaire for the past 12 months to take in account seasonal differences. The women were interviewed at home when they were about 28, 33 and 38 weeks pregnant. To estimate the portion sizes consumed we used models and pictures. The results showed a highly significant reduction in the intake of both whale meat and blubber, and blood analysis showed a corresponding reduction in the mercury exposure. However, the serum-PCB concentrations remained high as a possible consequence of slow elimination of these substances and perhaps also the impact of other dietary exposure sources, e.g. seabirds. Blood samples from approximately 100 of the children were drawn at the age of 13 months for determination of vaccination antibodies shortly after the third routine vaccination.

### Cohort 5

The most recent cohort was born during an 18-month period between October 2007 and April 2009. The total of mother-child pairs included is 475 (73% of the eligible population). Blood was taken from the cord, while blood, hair, and milk were obtained from the mother. The analytical results show that mercury exposures have declined substantially, with the geometric means being 4.4 μg/L cord blood and 0.71 μg/g hair. The maximum maternal hair-mercury concentration was 6.3 μg/g. The organochlorine exposures have also begun to decline. The geometric mean for serum-PCB was 0.42 μg/g lipid, with a maximum is 2.97 μg/g. In connection with the birth cohort formation, detailed data were obtained about the time to pregnancy and obstetrical parameters. In addition, 281 of the fathers participated in an examination of sperm quality along with a blood sample for organochlorine analysis. In addition, both grandmothers were invited for a dietary questionnaire and blood sampling for organochlorine analyses – as a possible reflection of prenatal exposure levels of the birth cohort parents. A total of 343 maternal and 206 of the paternal grandmothers participated. The children have are been examined at age 18 months, with more than 3 out of 4 contributing a blood sample. Follow-up will include a 42-month examination and detailed clinical examinations at 5 years of age, with emphasis on immunological parameters.

### Septuagenarians

To complement the birth cohorts, studies are also being carried out to examine the health status of elderly Faroese residents in regard to their life-time exposure to marine pollutants. In this population, where the contaminants mainly originate from pilot whale blubber and meat, past exposures can be estimated from dietary questionnaires. Persistent substances can also be measured in serum. A cohort of 1131 Faroese residents aged 70-74 years was therefore formed. The oldest subjects were invited first for the examinations, which took place at the National Hospital in Tórshavn over a 12-month period. A total of 713 subjects were examined (64% of the eligible population, excluding 14 deceased). Up to six septuagenarians per day arrived at the clinic before breakfast and, as the first procedure, fasting blood samples were obtained. All subjects then underwent a thorough physical examination, with a focus on neurobehavioral and cardiovascular function, as well as body weight, diabetes, and general health. Birth weights were extracted from the midwife charts kept at the Faroese National Archives. Cumulated exposures to major marine contaminants were assessed from analysis of blood samples. Serum was analyzed for persistent organochlorine pollutants. Current health and past medical history, including medication, were recorded by structured interview. A dietary questionnaire was used to ascertain the intake of traditional and other food during childhood and adolescence, adulthood, and the most recent year. The questionnaire focused on the amount of local food items, such as fish, whale meat and blubber, and seabirds in the diet. Other risk factors of possible relevance (such as smoking and alcohol use, and body weight at age 20) were also recorded.

## Results

The results of the above mentioned studies together with studies on the adult populations have revealed adverse health effects that are caused by contaminants in pilot whale meat and blubber:

### Mercury from pilot whale meat adversely affects the foetal development of the nervous system

Decrements in attention, language, verbal memory, and, to a lesser extent, in motor speed and visuospatial function, were associated with the mercury exposure. This association was still evident after the exclusion of high exposure subjects. As an objective neuophysiologial parameter, delays on brainstem auditory-evoked potentials were also associated with increased exposures. Exposure-related decrease in heart rate variability and a tendency of increased blood pressure were also found. Findings were replicated at age 14 years, when cohort members were examined by comparable methods. Adjustment for polychlorinated biphenyls exposure did not materially affect the mercury effects [1-6].

### The contaminants of the blubber adversely affect the immune system so that the children react more poorly to immunizations

A total of 587 children participated in the examinations at ages 5 and/or 7 years. At age 5 years, before the booster vaccination, the anti-diphtheria antibody concentration was inversely associated with PCB concentrations in milk and 18-month serum. Results obtained two years later showed an inverse association of concentrations of antibodies against both toxoids with PCB concentrations at age 18 months; the strongest associations suggested a decrease in the antibody concentration by about 20% for each doubling in PCB exposure. At age 5 years, the odds of an antidiphtheria antibody concentrations below a clinically protective level of 0.1 IU/L increased by about 30% for a doubling in PCB in milk and 18-month serum. In conclusions developmental PCB exposure is associated with immunotoxic effects on serum concentrations of specific antibodies against diphtheria and tetanus vaccinations. The immune system development during the first years of life appears to be particularly vulnerable to this exposure [7-9].

### Contaminants in pilot whales appear to increase the risk of developing Parkinson’s disease in those who often eat pilot whale

The study aimed to investigate the association of Parkinson’s disease (PD) with dietary exposure to polychlorinated biphenyls (PCBs) and methylmercury (MeHg) in a community with increased exposure levels. A total of 79 clinically verified idiopathic PD cases and 154 controls matched by sex and age were examined in this case–control study in the Faroe Islands. Blood and hair samples were collected and a questionnaire recorded lifetime information on residence, dietary habits, smoking history, and occupational exposure to solvents, pesticides, and metals. Both unconditional and conditional logistic regression analyses were used to estimate the odds ratio (OR) and 95% confidence interval (CI) in regard to relevant exposure variables. Increased ORs for dietary intakes of whale meat and blubber during adult life were statistically significant. The ORs for occupational exposure to solvents, pesticides and metals also suggested an increased risk for PD. Current serum concentrations of PCB and related contaminants suggested slightly increased ORs, although only b-hexachlorocyclohexane (b-HCH) was statistically significant. Increased intake of whale meat and blubber in adult life was significantly associated with PD, thus suggesting a positive association between previous exposure to marine food contaminants and development of PD [10].

### The risk of hypertension and arteriosclerosis of the carotid arteries is increased in adults who have an increased exposure to mercury [12]

We examined 42 male members (aged 30-70 years) of the Faroese whaling society to assess possible adverse effects within a wide range of methylmercury exposures from consumption of pilot whale meat. Exposures were assessed from mercury analysis of toenails, scalp hair, and whole blood, including a hair sample collected five years previously. Physiological measures included heart rate variability (HRV), blood pressures, common carotid intima-media thickness (IMT), and brainstem auditory evoked potentials (BAEPs). Structural equation analysis was carried out with independent exposure variables and outcome groupings, with adjustment for confounders, to determine the overall effect of mercury exposure on outcomes. Because of high correlations among related measures, the outcome groups consisted of the HRV parameters, diastolic blood pressure and heart rate, and BAEP results. The predictive validity of individual exposure biomarkers and a latent exposure variable on the individual outcomes was compared by multiple regression analysis. The results support the notion that methylmercury exposure promotes the development of cardiovascular disease, as expression by increased blood pressures and IMT [11].

### Marine food pollutants as a risk factor for hypoinsulinemia and type 2 diabetes

Clinical examinations of 713 Faroese residents aged 70–74 years (64% of eligible population) included fasting plasma concentrations of glucose and insulin, and glycosylated hemoglobin. Lifetime exposure to persistent environmental chemicals from pilot whale and other traditional food was estimated from a dietary questionnaire and by analysis of blood samples for polychlorinated biphenyls (PCBs) and related food contaminants. Septuagenarians with type 2 diabetes or impaired fasting glycemia tended to have higher PCB concentrations and higher past intake of traditional foods, especially during childhood and adolescence. In nondiabetic subjects, the fasting insulin concentration decreased by 7% for each doubling of the PCB concentration after adjustment for sex and body mass index at age 20. Conversely, the fasting glucose concentration increased by 6% for each doubling in PCB. Similar associations were seen in subjects without impaired fasting glycemia, while further adjustment for current body mass index and lipid metabolism parameters attenuated some of the associations. In conclusion impaired insulin secretion appears to constitute an important part of the type 2 diabetes pathogenesis associated with exposure to persistent lipophilic food contaminants [12].

## Perspectives

Recent risk assessments add perspective to the current limits for mercury concentration in fish. Because of beneficial nutrients, two seafood dinners per week is generally recommended as part of a varied diet. Two dinners would represent up to about 500 g of seafood. The reference dose determined by the U.S.EPA limit indicates that an adult (weight 70 kg) should not exceed a weekly mercury intake of 50 µg. This means that the seafood should contain an average mercury concentration of no more than 0.1µg/g. However, pilot whale meat contains in average 20 times as much [13].

According to the precautionary principle expressed in the Faroese Statement the medical authorities in the Faroe Islands have recommended to the government that pilot whale be considered unsafe for human consumption [14]. An allowance of a few grams per day in order to remain below intake levels considered safe is simply not realistic. It is ironic the this remote archipelago, which is not responsible for any significant mercury pollution, must now give up a traditional food source, which has contributed energy and essential nutrients to the population for many centuries.

## References

[B1] Grandjean P Weihe P White RF Debes F Araki S Yokoyama K Murata K Sorensen N Dahl R Jorgensen PJ Cognitive deficit in 7-year-old children with prenatal exposure to methylmercury Neurotoxicol Teratol 1997 19 6 417 428 10.1016/S0892-0362(97)00097-4 9392777

[B2] Steuerwald U Weihe P Jørgensen P Bjerve K Brock J Heinzow B Budtz-Jørgensen E Grandjean P Maternal seafood diet, methylmercury exposure, and neonatal neurologic function J Pediatr 2000 136 599 605 10.1067/mpd.2000.102774 10802490

[B3] Grandjean P Weihe P Burse VW Needham LL Storr-Hansen E Heinzow B Debes F Murata K Simonsen H Ellefsen P Budtz-Jørgensen E Keiding N White RF Neurobehavioral deficits associated with PCB 7-year-old children prenatally exposed to seafood neurotoxicants Neurotoxicol Teratol 2001 23 305 317 10.1016/S0892-0362(01)00155-6 11485834

[B4] Debes F Budtz-Jorgensen E Weihe P White RF Grandjean P Impact of prenatal methylmercury exposure on neurobehavioral function at age 14 years Neurotoxicol Teratol 2006 28 3 363 375 10.1016/j.ntt.2006.02.004 16647838 PMC1543702

[B5] Murata K Weihe P Budtz-Jorgensen E Jorgensen PJ Grandjean P Delayed brainstem auditory evoked potential latencies in 14-year-old children exposed to methylmercury J Pediatr 2004 144 2 177 183 10.1016/j.jpeds.2003.10.059 14760257

[B6] Sørensen N Murata K Budtz-Jorgensen E Weihe P Grandjean P Prenatal methylmercury exposure as a cardiovascular risk factor at seven years of age Epidemiology 1999 10 4 370 375 10.1097/00001648-199907000-00006 10401870

[B7] Heilmann C Grandjean P Weihe P Nielsen F Budtz-Jorgensen E Reduced antibody responses to vaccinations in children exposed to polychlorinated biphenyls PLoS Med 2006 3 8 e311 10.1371/journal.pmed.0030311 16942395 PMC1551916

[B8] Heilmann C Budtz-Jørgensen E Nielsen F Heinzow B Weihe P Grandjean P Serum concentrations of antibodies against vaccine toxoids in children exposed perinatally to immunotoxicants Environ Health Perspect 2010 118 10 1434 1438 10.1289/ehp.1001975 20562056 PMC2957925

[B9] Grandjean P Poulsen LK Heilmann C Steuerwald U Weihe P Allergy and sensitization during childhood associated with prenatal and lactational exposure to marine pollutants Environ Health Perspect 2010 118 10 1429 1433 10.1289/ehp.1002289 20562055 PMC2957924

[B10] Petersen MS Halling J Bech F Wernuth L Weihe P Nielsen F Jørgensen PJ Budtz-Jørgensen E Grandjean P Impact of diatary exposure to food contaminants on the risk of Parkinson’s disease Neurotoxicology 2008 29 4 584 590 10.1016/j.neuro.2008.03.001 18455239

[B11] Choi AL Weihe P Budtz-Jørgensen E Jørgensen PJ Salonen JT Tuomainen TP Murata K Nielsen HP Petersen MS Askham J Grandjean P Methylmercury exposure and adverse cardiovascular effects in Faroese whaling men Environ Health Perspect 2009 117 3 367 372 19337510 10.1289/ehp.11608 PMC2661905

[B12] Grandjean P Henriksen JE Choi AL Petersen MS Dalgård C Nielsen F Weihe P Marine food pollutants as a risk factor for hypoinsulinemia and type 2 diabetes Epidemiology 2011 22 3 410 417 10.1097/EDE.0b013e318212fab9 21364465 PMC3107006

[B13] National Research Council Toxicologial Effects of Methylmercury 2000 Washington DC: National Academy Press

[B14] Grandjean P Bellinger D Bergman Å Cordier D Davey-Smith G Eskenazi B Gray K Hanson M van den Hazel P Heindel JJ Heinzow B Hertz-Picciotto I Hu H Huang T T-K Jensen TK Landrigan PJ McMillen IC Murata K Ritz B Schoeters G Skakkebæk NE Skerfving S Weihe P The faroes statement: human health effects of developmental exposure to chemicals in our environment Basic Clin Pharmacol Toxicol 2008 102 2 73 75 18226057 10.1111/j.1742-7843.2007.00114.x

